# Genetic Diversity, Population Structure and Phylogeny of Indigenous Goats of Mongolia Revealed by SNP Genotyping

**DOI:** 10.3390/ani12030221

**Published:** 2022-01-18

**Authors:** Vera Mukhina, Gulnara Svishcheva, Valery Voronkova, Yurii Stolpovsky, Aleksei Piskunov

**Affiliations:** 1 Vavilov Institute of General Genetics, Russian Academy of Sciences, 119333 Moscow, Russia; atgc@vigg.ru (V.M.); gulsvi@mail.ru (G.S.); valery.voronkova@gmail.com (V.V.); Stolpovsky@mail.ru (Y.S.); 2 Institute for Information Transmission Problems, Russian Academy of Sciences, 127051 Moscow, Russia; 3 Institute of Cytology and Genetics, Siberian Branch of the Russian Academy of Sciences, 630090 Novosibirsk, Russia

**Keywords:** SNP genotyping, Mongolian goats, breed, conservational genetics

## Abstract

**Simple Summary:**

We performed SNP genotyping of indigenous goats of Mongolia to explore their demographic history in the global context and to estimate their genetic risks. Recently, these risks have become a subject of concern due to recent climatic disasters and uncontrolled massive breeding. Various clustering methods demonstrated close genetic relations among Mongolian, Russian, Chinese, and West Asian breeds. Mongolian goats themselves exhibited low to moderate estimates of genetic differentiation. We identified genetic features highlighting the distinct origin and breeding history of Mongolian goat breeds, as well as traces of artificial selection and geographic isolation. However, none of them met formal criteria to be considered as endangered.

**Abstract:**

Mongolian goats are of great interest for studying ancient migration routes and domestication, and also represent a good model of adaptability to harsh environments. Recent climatic disasters and uncontrolled massive breeding endangered the valuable genetic resources of Mongolian goats and raised the question of their conservation status. Meanwhile, Mongolian goats have never been studied on genomic scale. We used Illumina Goat SNP50 to estimate genetic risks in five Mongolian goat breeds (Buural, Ulgii Red, Gobi GS, Erchim, Dorgon) and explored phylogenic relationships among these populations and in the context of other breeds. Various clustering methods showed that Mongolian goats grouped with other Asian breeds and were especially close to some neighboring Russian and Chinese breeds. The Buural breed showed the lowest estimates of inbreeding and exhibited the shortest genetic distances within the other Mongolian breeds, especially, to Ulgii Red and Gobi GS. These three breeds formed a single core group, being weakly differentiated from each other. Among them, Gobi GS displayed obvious signs of inbreeding probably resulted from artificial selection pressure. Dorgon and especially Erchim goats stand apart from the other Mongolian breeds according to various types of analyses, and bear unique features pointing to different breeding histories or distinct origins of these breeds. All populations showed strong decline in effective population size. However, none of them met formal criteria to be considered as endangered breeds. The SNP data obtained in this study improved the knowledge of Mongolian goat breeds and could be used in future management decisions in order to preserve their genetic diversity.

## 1. Introduction

Although the conservation of biodiversity is now recognized as one of the main focuses in genetic research in domesticated animals, the great efforts made here still cannot fix the rate of loss in local animal breeds [1]. Goat breeds are mainly distributed in developing countries, providing essential support for the huge part of the population, while the global demand for goat products accelerates an intensification of goat farming occurs and promotes an invasion of transboundary breeds in order to increase productivity. Adaptability of local goats serves as the main source of stability for small holders when faced with environmental changes [2], so that loss in genetic diversity puts a number of cultures and ethnic groups under the threat of extinction. For goats, biodiversity conservation is particularly challenging because the number of their breeds is huge, but conservation resources are very limited. In comparison with other domesticated animals, the biodiversity of goats is the least characterized [3,4]. Thus, the primary challenge today is to identify breeds that need to be conserved first. Decisions might be supported by the compilation of databases containing objective information about the genotypes of these breeds: their genetic risks, phylogenetic relationships, as well as knowledge of the socioeconomic processes that can affect the risks of these breeds [5,6]. SNP genotyping is widely recognized as the most appropriate tool due to the availability of commercial SNP arrays that give unified comparable data [7].

In Mongolia, goats have been the main source of food and clothing for several thousand years. Some authors believe that independent goat domestication could have happened in Mongolia [8]. Local goats are adapted to year-round grazing in extreme climates and altitudes of the Altai Mountains, steppes, and Gobi Desert. In some areas, differences between summer and winter temperatures exceed 100 °C. In certain regions, local goats can satisfy their nutritional demands by finding plants under the snow.

Mongolian goats have experienced various perturbations over the past decades. With the fall of the Soviet Union, the Mongolian market became open to the rest of the world. Therein, the goat population has increased several times as a result of the global demand for cashmere [8]. This increase gives a false impression of the industry’s well-being. Meanwhile, farmers still rely on productive transboundary breeds as a source of improving the quality of their local goats, which can destabilize goats’ adaptation traits. Moreover, a series of climatic disasters caused repeated reductions in goat populations, which could have cause bottlenecks and increased inbreeding. According to the National Statistical Office of Mongolia, 11.5 million livestock animals perished during the winters of 1999–2002, and the harsh winter of 2009–2010 killed 10.3 million animals [9]. There is no reliable information whether these disasters affect mainly local or invasive breeds.

Meanwhile, Mongolian goats’ genetic diversity, phylogeny among local breeds, and their relations with breeds found in the rest of the world are still largely unknown. The largest unified dataset of goat genotypes provided by the Adaptmap project covers many areas all around the world, but completely lacks samples from Central and East Asia [10]. These regions are key to understanding history of goat domestication and migration, but they are represented only by small, disjointed datasets. In particular, we are not aware of any studies of Mongolian goats that used SNP genotyping. There are few studies that examined diversity of Mongolian breeds by STR, mtDNA, and Y-chromosome genes analysis between 2003 and 2020 [8,11,12]. All of these studies concluded that local Mongolian goat breeds are weakly differentiated genetically, but no thorough analysis of population structure and phylogeny in context of other breeds was done.

Using SNP genotypes, we examined demographic history and estimated genetic diversity of indigenous goats of Mongolia and explored their phylogenetic relationship with goats of the rest of the world.

## 2. Materials and Methods

### 2.1. Sample Collection and Description

**Figure 1:**
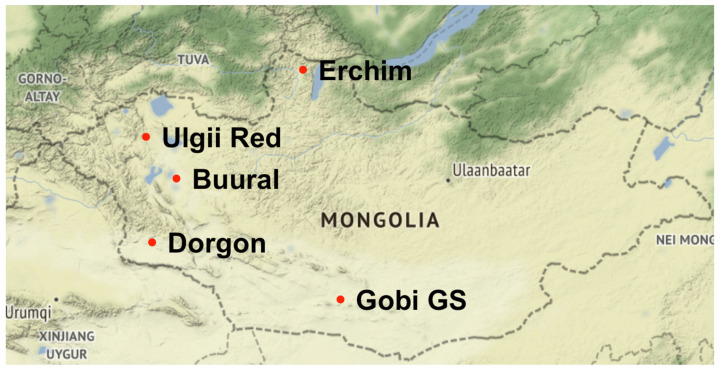
Map of sampled populations.

Blood was sampled from 244 animals in five Mongolian goat populations located in five geographical areas of the country with different climatic conditions during a research expedition of the Vavilov Institute of general genetics in 2017, one breed per location. The geographical locations of sampled populations and brief sample descriptions are summarized in Figure 1 and Table 1.

One of the peculiarities of Mongolian goats is the lack of deep specialization of their productivity: dairy, downy, or meat ones, which makes them versatile. All the breeds except for the Gobi Gurvan Saikhan (Gobi GS) were formed mostly by natural selection and adapted to round-year grazing and nomadic breeding style. Gobi GS was developed by crossing local cashmere goats in the Gobi area with Don breed bucks up to the F2, followed by pure and selective breeding [13]. Buural goats are the most widely distributed breed in Mongolia, whereas Erchim goats of the Darkhat Valley are geographically isolated local populations [14].

### 2.2. DNA Extraction and Genotyping

DNA was extracted from the whole blood samples containing EDTA as an anticoagulant using Magna Prep 200 kit (Laboratory Isogen, Moscow, Russia) to the manufacturer’s instructions. Quality of the DNA was measured using the Nanodrop 8000 spectrophotometer. Genotyping and preliminary data analysis were performed by Federal Center for Animal Husbandry (Russia). Genotyping was performed with Goat 50K BeadChip (Illumina Inc., San Diego, CA, USA) containing 50,347 SNPs. Quality control was performed by setting a cutoff of 0.5 for the GenCall and GenTrain scores. The SNP dataset was filtered to remove poorly genotyped individuals, loci genotyped in <90% of individuals, and rare alleles using PLINK 1.9 [15] with --geno 0.1 --mind 0.1 --maf 0.05 plink parameters. To ensure that our analysis would not be affected by the presence of SNP in strong linkage disequilibrium, we also filtered data with --indep-pairwise 50 5 0.2 PLINK filter; 184 samples passed these filters. Among highly related pairs of animals, we selected only one animal per pair for future analysis, thus 17 more animals were excluded. A total of 167 samples were processed in the future analysis.

### 2.3. Construction of the Working Datasets

To explore relationships between Mongolian populations and breeds from the rest of the world, we added genotype data obtained in other studies (Table S1) to our dataset. First, we added data from the AdaptMap project described in the paper by Colli et al. [10] that contained goat population data from all over the world. Asia was underrepresented in this dataset. There were just a few populations from West Asia (Iran, Turkey, and Pakistan), but no data from the countries located close to Mongolia. To fill this gap, we added genotypes from the study by Deniskova et al. [16] presenting seven goat breeds from Russia, whereas three of them were collected close to Mongolian border (Altai Mountain (ALTM), Altai White Downy (ALTW), Soviet Mohair (SOVM)) and one near the Kazakhstan border (Orenburg (OREN)). We also added data from six populations from five Chinese regions described by Berihulay et al. [17]. Among them, Nanjiang (NJ) and Qinggeda (QG) breeds were sampled in the region next to the southwest Mongolian border, and Arbas Cashmere (AC) was sampled near the southern border.

Raw datasets were combined using --merge PLINK command and filtered with --geno 0.01 --mind 0.2 --maf 0.001 parameters. --mind rate was set to be much more relaxed than usual to keep relatively poorly genotyped Mongolian, Russian, and Chinese goats in the analysis. To ensure that analyses would not be distorted by the presence of SNPs in a strong linkage disequilibrium (LD), the --indep-pairwise 50 5 0.1 command in PLINK was used to prune the SNPs that passed the initial filtering step.

To obtain datasets with different resolution, we further created four subsets, namely, Worldwide, Asian, Local, and Mongolian. The Worldwide dataset included all available samples. The Asian dataset included goats from China, Mongolia, West Asia, and all Russian samples, except the transboundary Saanen (SAAN) breed. The Local dataset included Mongolian, Nanjiang, Qinggeda, and Orenburg samples, whereas the Mongolian dataset consisted exclusively of the SNP data obtained in this study. The three additional breeds included in the Local dataset were chosen because of their geographic proximity and genetic closeness revealed by principal component and phylogenetic analyses.

### 2.4. Genetic Diversity Estimation

To evaluate within-population genetic diversity, we calculated the observed heterozygosity (Ho), unbiased expected heterozygosity (He), and inbreeding coefficient (Fis) with 95% confidence intervals (CI 95%) using the R package “diveRsity” [18].

#### 2.4.1. Effective Population Sizes

Trends in effective population size were estimated by linkage disequilibrium (LD) using SNeP tool [19] with default parameters, except the ones for the sample size correction, occurrence of mutation (α = 2.2) [20], and recombination rate between a pair of genetic markers according to Sved and Feldman [21].

#### 2.4.2. Runs of Homozygosity (ROH)

ROH analysis was performed for the Mongolian dataset without LD and minor allele frequency pruning. To calculate ROH, we used the methodology of Meyermans et al. [22] with settings suitable for medium-density genotype samples (PLINK commands: --homozyg-density 50 --homozyg-kb 1000 --homozyg-snp 20 --homozyg-window-het 1 --homozyg-window-snp 20). Non-parametric Kruskal–Wallis test followed by Dunn test with Benjamini–Hochberg correction (*p*-value cutoff = 0.05) for multiple testing from dunn.test R package [23] were used to estimate differences in ROH lengths and frequencies among breeds.

The genomic inbreeding coefficient based on ROH (F_ROH_) was computed as the sum of the length of all ROH per goat as a proportion of the total autosomal SNP coverage.

#### 2.4.3. Haplotype Sharing

To estimate an impact of recent admixture events, we performed an analysis of haplotype sharing. To phase our Worldwide dataset, we used ShapeIT software [24] with default parameters and then performed sharing analysis with Refined IBD [25] with default conditions. Results were processed and visualized in R.

### 2.5. Genetic Relationships and Population Structure

To investigate genetic relationships among Mongolian and the other breeds, we performed both principal component (PCA) and phylogenetic analyses. PCA was performed using PLINK and was further visualized in R. The individual-level phylogenetic analysis of the Local dataset was performed using the aboot() function from the R ‘poppr’ package [26]. Dendrogram (with 1000 bootstrap-support replicates) were built using Hamming (bitwise) genetic distances and the neighbor joining algorithm.

Genomic clustering of Worldwide and Local datasets was performed using fastSTRUCTURE software [27]. The program runs were carried out assuming the K-value to be between 2 and 15 groups in 50 repeats. The cluster membership matrices of the fastSTRUCTURE outputs were visualized using PONG software [28].

Pairwise F_ST_ values [29] were calculated for phylogenetic studies in the R package diveRsity [18]. The matrix of pairwise F_ST_ values was visualized as a Neighbor Net group genetic network using SplitsTree 4.14.5 software [30].

## 3. Results

### 3.1. Dataset Description

We generated a set of 45,665 SNPs from 167 animals from five Mongolian breeds (Buural, Ulgii Red, Erchim, Gobi GS, Dorgon). To analyze relationships of these goats with other breeds we added data from three previously published datasets from Russia, China, and other countries [10,16,17]. This combined dataset, the Worldwide dataset, contained 38,276 SNPs from 5176 animals of 151 breeds (Table S1). Additionally, we subsetted 39 breeds from Russia, Mongolia, China, Iran, Turkey, and Pakistan into the Asian dataset.

### 3.2. Genetic Diversity and Effective Population Sizes in Mongolian Populations

To estimate genetic diversity, we calculated the observed (Ho) and unbiased expected heterozygosity (He) (Table 2). According to Ho and He values, Erchim goats exhibited the minimum level of genetic diversity, and Buural goats showed the maximum. For all Mongolian populations, except Gobi GS, observed heterozygosity did not differ from expected values by more than 0.005. The Gobi GS breed showed the highest difference between Ho and He that equaled 0.010. Such high difference could be due to inbreeding having occurred during breed development. This hypothesis was also supported by relatively high inbreeding coefficient value (Fis) for this breed. The Erchim goats showed an excess of heterozygotes according to negative Fis values.

**Figure 2:**
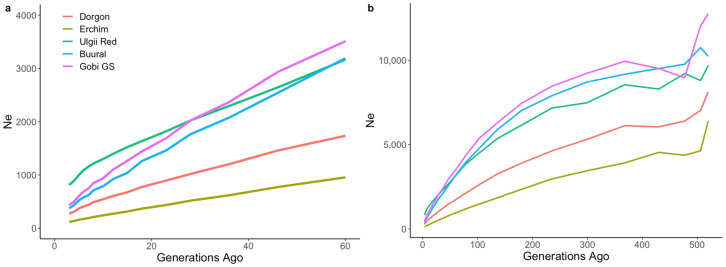
Recent (**a**) and long-term (**b**) trends in effective population sizes (Ne) for the five Mongolian goat populations.

Ancestral and recent effective population sizes (Ne) for five Mongolian goat populations are shown in Figure 2. Estimated Ne showed a downward trend with the increase in generations across all populations. The Erchim goat breed had the lowest effective population size at all time points, decreasing from 955 to 120 animals over the last 60 generations. Recent effective numbers for other breeds were 274 for Dorgon, 373 for Buural, 433 for Gobi GS, and 812 for Ulgii Red. The steepest decline in the effective population size was detected in the Gobi GS breed.

### 3.3. Runs of Homozygosity (ROH)

**Figure 3:**
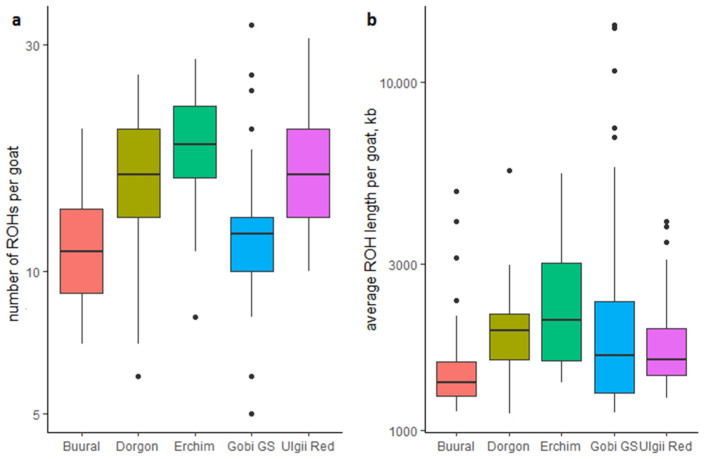
Distributions of ROH frequencies (**a**) and their average lengths (**b**) across Mongolian breeds.

To estimate the level of inbreeding, we calculated the ROH lengths for each Mongolian goat. Every recombination event reduces the ROH length, thus the longer ROHs mark recent inbreeding events. The most variable ROH lengths were found in the Gobi GS breed (Figure 3 and Figure S1) with some outliers exhibiting a high amount of extremely long ROHs, whereas other goats were particularly poor in ROHs. These observations suggested that some Gobi GS goats were highly inbred. Given the historical background of the breed, which originated from crossbreeding of local goats with the Russian Don breed, the inbreeding was likely to be a result of artificial selection pressure.

The average ROH length was significantly lower in Buural goats compared to all other breeds (Dunn test, *p*-value < 0.05). Furthermore, significant deficit in ROH frequency was observed in this breed compared to Dorgon, Erchim, and Ulgii Red breeds. The average ROH length in Erchim goats was significantly higher compared to other breeds except Dorgon. All breeds display low average F_ROH_ values ranging from 0.007 in Buural to 0.023 in Gobi GS (Table 2), whereas some Gobi GS outliers demonstrate individual F_ROH_ values around 0.2.

### 3.4. Ancestry of Mongolian Goat Breeds

#### 3.4.1. Principal Component Analysis

**Figure 4:**
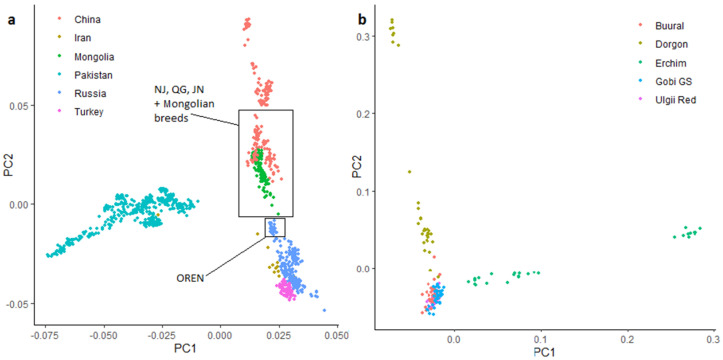
Principal component analysis for all goats of Asian origin (**a**) and Mongolian breeds (**b**). OREN = Orenburg, NJ = Nanjiang, QG = Qinggeda, JN = Jining Grey.

Principal component analysis showed that goat populations clustered in agreement with their geography and domestication history (Figure 4, Figure S2 and Figure S3). Chinese, Mongolian, most West Asian (Iran, Pakistan, Turkey) goats and goats from the Asian part of Russia formed a single cluster separately from samples of the other regions. On the detailed PCA graph for Asian breeds (Figure 4a and Figure S3), only the first component (PC1) clearly separated Pakistani populations from the rest: Chinese, Mongolian, Russian, Turkish, and Iranian breeds. Mongolian Dorgon, Erchim, and Ulgii Red breeds were clustered together with Chinese Nanjiang, Qinggeda, and Jining Grey breeds (Figure S3), whereas Gobi GS goats tended towards the Orenburg breed from Russia.

The PCA of Mongolian breeds revealed a low differentiation within Buural, Gobi GS, and Ulgii Red breeds, but high variability within Erchim and Dorgon breeds (Figure 4b). Gobi GS, Buural and Ulgii Red samples were plotted together in the single cluster, and Erchim and Dorgon breeds were separated by the first and the second principal components, respectively. Both breeds formed two clusters each: one cluster was placed quite close to the other Mongolian samples, whereas the second one was clearly differentiated.

#### 3.4.2. FastSTRUCTURE Analysis

**Figure 5:**
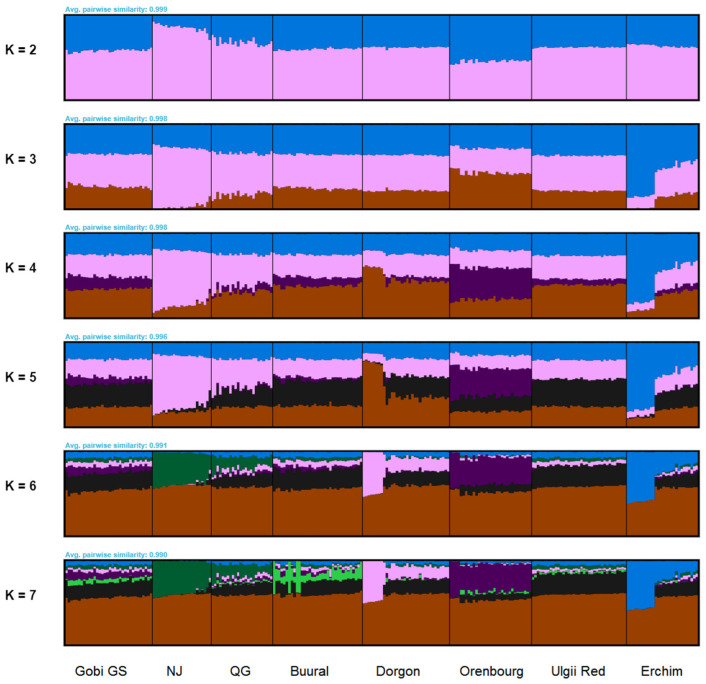
FastSTRUCTURE clustering for the Local dataset. NJ = Nanjiang, QG = Qinggeda.

The fastSTRUCTURE analysis applied to the Worldwide (Figure S4) and the Local datasets (Figure 5) showed results similar to the PCA results. Mongolian breeds displayed low differentiation among the breeds and clustered together with Chinese, most Russian and West Asian breeds (Figure 5). A small admixture of European and transboundary Saanen breeds was observed as well as with Angora goats (Figure S4).

Local fastSTRUCTURE revealed non-uniform structure of two goat populations, Erchim and Dorgon (Figure 5). Nearly half of the samples of these populations clearly formed their own separate clusters and showed reduced presence of other components uniformly distributed in the rest of Mongolian goats. Consistent with PCA results, the genetic pool of Orenburg goats influenced the Gobi GS population. The most likely number of populations according to the maximum likelihood estimation was equal to 5.

#### 3.4.3. Phylogenetic Analysis

**Figure 6:**
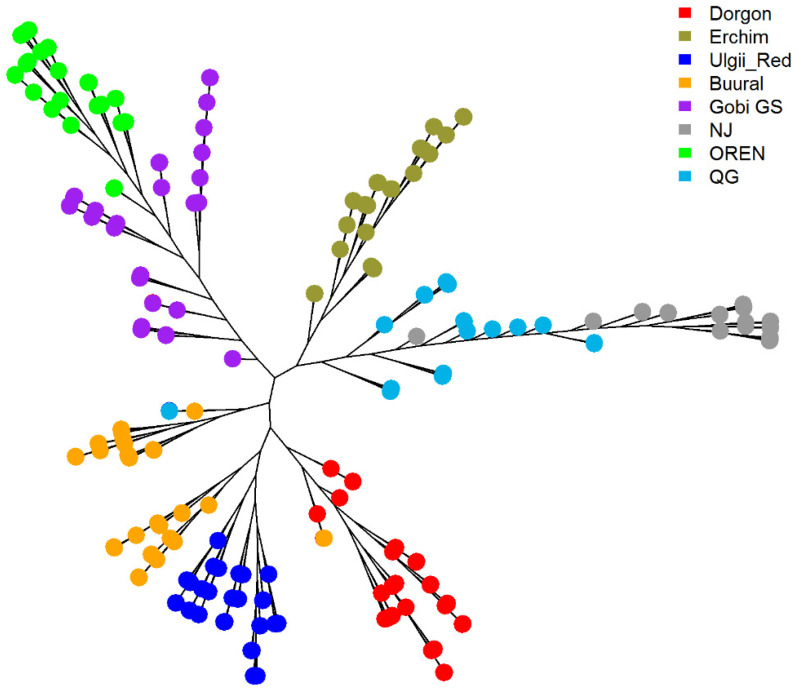
Neighbor joining phylogenetic tree for the Local dataset: Mongolian breeds, two Chinese breeds (Nanjiang, NJ, and Qinggeda, QG), and one Russian breed (Orenburg, OREN).

We constructed an individual-level neighbor joining phylogenetic tree for the Local dataset including Mongolian samples, two Chinese breeds (Nanjiang, and Qinggeda), and one Russian (Orenburg) breed (Figure 6). For easier visualization, we did not include bootstrap values for the pairs of individuals in Figure 6. Gobi GS formed a single cluster with Orenburg goats confirming the fastSTRUCTURE results. The Erchim goats of the Darkhat Valley were most closely related to Chinese breeds. Dorgon and Ulgii Red goats formed a single cluster closely related to 16 Buural goats. Whereas the rest, 19 Buural goats, were related to Gobi GS goats, also confirming PCA results. Nanjiang and Qinggeda breeds’ positions resembled those in the original paper [17].

#### 3.4.4. Genetic Distances (F_ST_)

**Figure 7:**
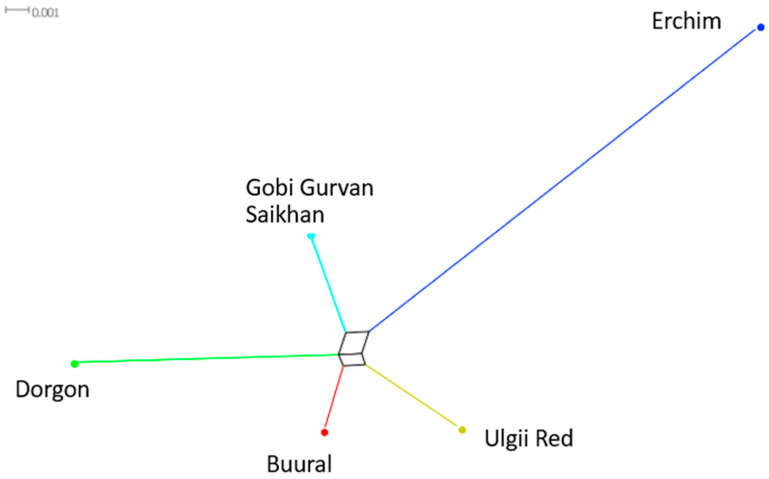
F_ST_-based Neighbor-Net tree for Mongolian goats.

The genetic distances (F_ST_) (Table 3) and the corresponding Neighbor-Net tree (Figure 7) showed Erchim to be the most genetically autonomous breed among the Mongolian goats in our study. The closest genetic distances were found in the pairs of Buural-Gobi GS and Buural-Ulgi Red.

#### 3.4.5. Haplotype Sharing

**Figure 8:**
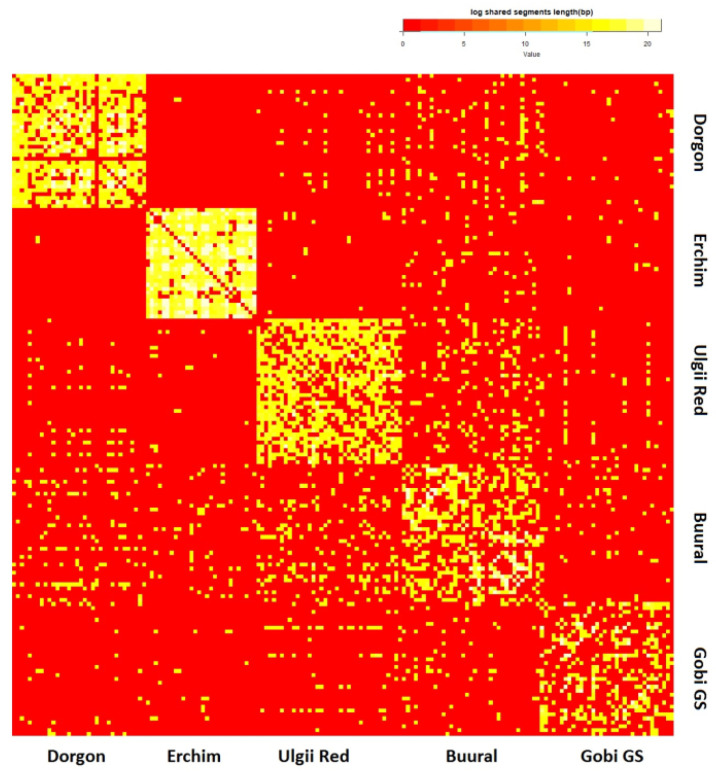
Heatmap: haplotype sharing (>7 Mb) within Mongolian goat populations (by individuals). Yellow indicates shared haplotypes.

**Figure 9:**
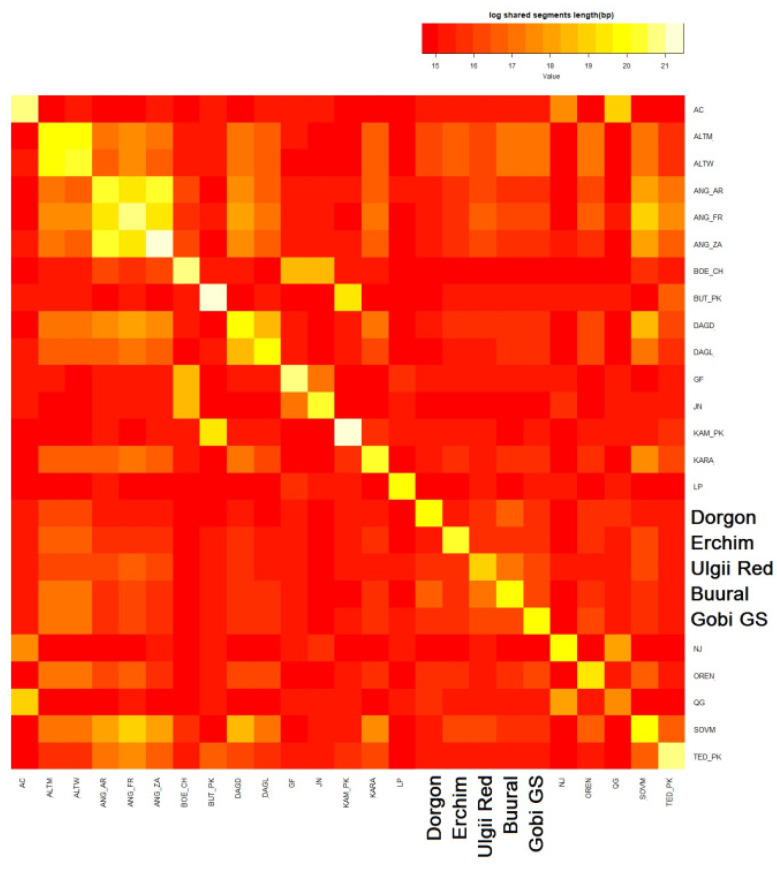
Heatmap: haplotype sharing within Mongolian goats and 20 most closely related breeds (samples detailed in Table S1).

To explore recent admixture events, we analyzed haplotype sharing in Mongolian goat populations between each other (Figure 8) and in comparison with the top-20 related goat breeds (Figure 9).

According to Figure 8, the Buural breed was the most haplotype-sharing population in our study, followed by the Ulgii Red breed. These two populations had the most intensive haplotype sharing between each other, appearing almost like a single population on the heatmap. The Erchim goats evidently had the lowest proportion of shared haplotypes across Mongolian populations consistent with the geographic isolation of this breed.

When plotted within the 20 most closely related breeds, all of the Mongolian goats had the highest proportion of shared haplotypes with Altai Mountain (ALTM), Altai White Downy (ATLW), Soviet Mohair (SOVM), Dagestan Downy (DAGD), and Angora (ANG_AR, ANG_FR, ANG_ ZA) goats. The Angora breed originated in Turkey and further became a transboundary breed and thus was involved in improvement of many local breeds; its impact on ALTM, SOVM, and DAGD breeds is well documented [16]. In contrast to the PCA results, Chinese Nanjiang and Qinggeda on the heatmap appeared unrelated to Mongolian goats. The Dorgon goats were the only one of Mongolian populations that showed a trace of haplotype exchange with Qinggeda (but not Nanjiang) goats.

## 4. Discussion

Genotyping by SNP arrays is an accurate and cost-efficient tool to analyze various indicators of genetic diversity. While large efforts are dedicated towards studying commercial high-yielding breeds, there is a growing interest in genetics of local populations due to their specific traits allowing them to adapt to local environments and resist pathogens, starvation, and harsh climatic conditions. There is a global trend to improve indigenous goats with transboundary breeds while trying to preserve useful local traits, thereby helping these admixed goats to remain adapted, especially, in such extreme regions as Mongolia. Moreover, some local breeds need to be conserved to maintain goat biodiversity and provide support to nomadic traditions and local communities, which are very typical for Mongolia. Whole genome SNP analysis allows exploring of genetic diversity and may help to achieve these goals.

Additionally, goat genotypes from Central and East Asian regions could provide valuable information about ancient migration routes and domestication events. Meanwhile, these regions are covered unevenly by just few projects with low to moderate numbers of samples.

We performed the first whole genome SNP analysis of populations of Mongolian goats and integrated these data with other local and global datasets. Our study complemented the results obtained in previous works that used STR [11,31] and mtDNA [8,12,14] analyses. Consistent with our PCA and fastSTRUCTURE results, these studies also generally assumed weak differentiation across Mongolian goats [14]. Mongolian goats, apparently, historically were adapted to the semi-wild and nomadic way of life and therefore crossed more freely than worldwide populations. Thus, they may represent lower indices of differentiation.

All of the five goat populations of Mongolia showed a tendency in reducing effective population sizes, but none of the populations had Ne values decreased below the generally accepted limit of 100 or even 50 individuals to be considered as endangered [32]. However, these threshold values were mostly obtained by equational modeling [32] and are rarely verified empirically. Better modeling combined with empirical validation is expected to improve assessment of genetic risks based on Ne [33,34]. Decrease in ancestral population size, on the other hand, appears to be typical for most local breeds from all over the world [10], probably just meaning that domestication and breed development led to separation of larger populations [35]. Some subpopulations of commercial high-yielding breeds, such as Saanen or Angora, also exhibit small Ne values even below the threshold [16,36]. The most dramatic drop in Ne among Mongolian goats was observed in the Gobi GS breed, which is known to be under the highest artificial selection pressure among all goat populations in this study.

To put Mongolian goats in a broader context, we compared our results with previously reported data from other breeds and populations of the rest of the world. PCA partitioning of all Asian breeds into two clusters (Pakistani vs. others) probably resembles southern and northern goat dispersal routes in Asia [37]. After the primary domestication, one branch migrated through Caucasus and Siberia to China, and another one moved to Pakistan. In accordance with this scenario, the second component placed the Mongolian goats between two clusters that consisted mainly of Russian and Chinese samples. However, more samples from Southern and Central Asia are required to fill the gap and to add more support to this scenario. The PCA and fastSTRUCTURE, as well as phylogenetic analyses, demonstrated extensive common ancestry shared with Asian breeds, especially, with some Chinese, Altai, and Orenburg populations. We also detected a trace of recent admixture with Angora-like breeds, but not with Chinese goats, in most of Mongolian populations.

For Erchim goats, estimates of genetic distances (F_ST_) and various clustering methods showed the Erchim breed inhabiting the Darkhat Valley to be the most autonomous population among the Mongolian goats in our study. Origin of Erchim goats remains unclear. According to the PCA, they were more closely related to Chinese goats than to the rest of Mongolian or other Asian samples. Neighbor joining and Nei-distance trees also clustered them together with Chinese Nanjiang and Qinggeda goats. However, analyses of shared haplotypes revealed recent admixture events from the Altai goats. The fastSTRUCTURE analysis demonstrated two separate subpopulations within this breed. One of them looked very similar to the rest of the Mongolian breeds, but the another one bore genetic material unique to the local population. This partition could be explained by demographic reasons and may potentially have affected the results of the tests performed for the breeds as a whole (e.g., F_ST_ based tree or haplotype sharing with related breeds).

ROHs for Erchim goats were relatively long and frequent compared to other Mongolian breeds, but the F_ROH_ and Fis values indicated that they were not highly inbred. Furthermore, the Erchim goats exhibited the lowest effective population size that continued to decline but do not yet meet the criteria for endangered breeds. Taken together, our data have indicated that this breed requires special attention and further investigation in light of its autonomy, intricate population history, and adaptability to harsh conditions.

The Dorgon goats, as well as Erchim, were also in two subpopulations according to PCA and fastSTRUCTURE with one subpopulation demonstrating a unique genetic material potentially of distinct origin.

The Gobi GS breed was also a bit shifted on the PCA from the main pool of the Mongolian samples toward Orenburg goats. The study by Takahashi et al. [14] showed similar results, including separation of Erchim and Gobi GS goats by PCA analysis of eight microsatellite loci in eight Mongolian goat populations. Gobi GS is the only Mongolian breed that was created by classical intense artificial selection. Gobi GS is a crossbred with Russian Don breed [31]. The Don breed was officially recognized after an internal Soviet Union expedition in 1934. According to the Food and Agriculture Organization of the United Nations, animals of the breed probably produced the highest wool yield among documented goat breeds [1]. The Don goats were widely used for improvement of Orenburg and Altai Russian breeds. In turn, Angora goats originated from Ankara, the capital city of Turkey, located in the territory of ancient Anotalia, one of the regions of the Fertile Crescent. This breed was also widely used for improvement of many goat breeds around the world, including Australia and North America, and, more locally, for improvement of Soviet Mohair Russian goats [38]. The fact that Gobi GS goats in our study placed near the Orenburg breed is likely explained by an admixture of Don goats used while creating these breeds. Thus, shared genetic variance between Gobi GS and Orenburg breeds might represent a reservoir of the gene pool of Don goats, a breed that has never been characterized by SNP genotyping. The area on PCA that overlaps Gobi GS and Buural goats suggests the latter might be the Mongolian ancestor of the Gobi GS breed. Indeed, Buural is the predominant breed of goats in Mongolia that was widely used in local breeding.

Thus, the Buural goats were probably used in developing the Gobi GS breed, since these two breeds showed low genetic distances and were plotted closely on PCA. Furthermore, the Buural goats appeared to exhibit lower genetic autonomy as compared with other Mongolian breeds. These goats exhibited the lowest inbreeding estimates and the widest genetic relationships both within other Mongolian breeds and within world goat populations. These data make sense given the fact that Buural goats are the most widely distributed ones in Mongolia, at least among the studied populations [14].

Two breeds from Western Mongolia (Buural and Ulgii Red) were the most closely related breeds and clustered together in the most types of analyses. Together with the Gobi GS, they formed a core cluster of the studied Mongolian breeds with a weak genetic differentiation.

## 5. Conclusions

Our analysis of Mongolian goats revealed their close relationship to neighboring Chinese and Russian breeds. Our study demonstrated low genetic differentiation within Mongolian breeds, especially among Buural, Ulgii Red, and Gobi GS. These breeds formed a single core cluster, whereas Dorgon and, especially Erchim goats, were more autonomous. We identified genetic features highlighting distinct origin and breeding history in Mongolian goats and also indicating long-term preservation of specific allele combinations of ancestor breeds. All breeds showed strong tendency in reducing effective population sizes, but none of them yet meet the criteria for endangered breeds. Taken together, our analysis suggests Mongolian goats to be a valuable reservoir of genetic resources and provide useful information for the future investigations of goat diversity.

## Figures and Tables

**Table 1:** Sampled populations and their environments.

Breed (Code)	n ^1^	Prefecture	Coordinates	Environment	Areas of Benefit	Color
Erchim (EB)	28	Huvsgul	51.42, 99.73	Mountain areas with extreme continental climate	Milk, meat, and cashmere	Black
Dorgon (DG)	34	Hovd	45.80, 92.29	Western steppes and mountains	Cashmere, meat, and milk	White and red
Buural (ZB)	35	Zavkhan	47.95, 93.49	Gobi-like areas of Great Lake Valleys	Cashmere and milk	Dark brown and black
Ulgii Red (UR)	37	Uvs	49.31, 91.99	Both mountain and steppe areas	Cashmere, meat, and milk	Red and brown
Gob Gurvan Saikhan (GGS)	33	Gurvan Saikhan	43.80, 101.58	South Gobi, Three Beauties’ mountains	Cashmere	Various. Mostly red and white

^1^ Samples that passed quality controls and were further included in statistical analysis.

**Table 2:** Genetic diversity and inbreeding estimates in Mongolian goats by observed (Ho), unbiased expected heterozygosity (He), their difference (dH), estimates of the inbreeding coefficient (Fis) with the 95% confidence interval and mean F_ROH_.

Breed	Ho	He	dH	Fis	F_ROH_
Dorgon	0.396	0.395	0.001	−0.004 (−0.006; −0.002)	0.014
Buural	0.406	0.405	0.001	−0.002 (−0.004; 0.000)	0.007
Erchim	0.393	0.388	0.005	−0.013 (−0.015; −0.011)	0.019
Gobi GS	0.400	0.410	−0.010	0.023 (0.021; 0.025)	0.023
Ulgii Red	0.397	0.399	−0.002	0.004 (0.003; 0.005)	0.013

**Table 3:** The genetic distances (F_ST_) between Mongolian goats.

Breed	Dorgon	Buural	Erchim	Gobi GS	Ulgii Red
Dorgon					
Buural	0.015				
Erchim	0.035	0.027			
Gobi GS	0.017	0.009	0.027		
Ulgi Red	0.018	0.009	0.028	0.012	

Note: All pairwise F_ST_ values are significant (permuted *p*-values < 0.001 for all estimates).

## Data Availability

https://drive.google.com/file/d/1NUz7AO63NQRqwD34Jf-RGXzzJ4ezOrIt/view?usp=sharing.

## Supplementary Materials

The following supporting information can be downloaded at: https://www.mdpi.com/article/10.3390/ani12030221/s1, Figure S1: Distribution of ROH lengths and amount of ROHs; Figure S2: Principal component analysis for the Worldwide dataset; Figure S3: Proncipal component analysis for the Asian dataset; Figure S4: FastSTRUCTURE analusis for the Worldwide dataset; Table S1: Goats datasets.

## References

[1] Bélanger J., Pilling D. (2019). The State of the World’s Biodiversity for Food and Agriculture.

[2] Joy A., Dunshea F.R., Leury B.J., Clarke I.J., DiGiacomo K., Chauhan S.S. (2020). Resilience of small ruminants to climate change and increased environmental temperature: A review. Animals.

[3] Galal S. (2005). Biodiversity in goats. Small Rumin. Res..

[4] Olschewsky A., Hinrichs D. (2021). An Overview of the Use of Genotyping Techniques for Assessing Genetic Diversity in Local Farm Animal Breeds. Animals.

[5] Stella A., Nicolazzi E.L., Van Tassell C.P., Rothschild M.F., Colli L., Rosen B.D., Sonstegard T.S., Crepaldi P., Tosser-Klopp G., Joost S. (2018). AdaptMap: Exploring Goat Diversity and Adaptation.

[6] Cortellari M., Barbato M., Talenti A., Bionda A., Carta A., Ciampolini R., Ciani E., Crisà A., Frattini S., Lasagna E. (2021). The climatic and genetic heritage of Italian goat breeds with genomic SNP data. Sci. Rep..

[7] Pogorevc N., Simčič M., Khayatzadeh N., Sölkner J., Berger B., Bojkovski D., Zorc M., Dovč P., Medugorac I., Horvat S. (2021). Post-genotyping optimization of dataset formation could affect genetic diversity parameters: An example of analyses with alpine goat breeds. BMC Genom..

[8] Ganbold O., Lee S.-H., Paek W.K., Munkhbayar M., Seo D., Manjula P., Khujuu T., Purevee E., Lee J.H. (2020). Mitochondrial DNA variation and phylogeography of native Mongolian goats. Asian-Australas. J. Anim. Sci..

[9] Rao M.P., Davi N.K., D’Arrigo R.D., Skees J., Nachin B., Leland C., Lyon B., Wang S.-Y., Byambasuren O. (2015). Dzuds, Droughts, and Livestock Mortality in Mongolia. Environ. Res. Lett..

[10] Colli L., Milanesi M., Talenti A., Bertolini F., Chen M., Crisà A., Daly K.G., Del Corvo M., Guldbrandtsen B., Lenstra J.A. (2018). Genome-wide SNP profiling of worldwide goat populations reveals strong partitioning of diversity and highlights post-domestication migration routes. Genet. Sel. Evol..

[11] Beketov S., Piskunov A., Voronkova V., Petrov S., Kharzinova V., Dotsev A., Zinovieva N., Selionova M., Stolpovsky Y.A. (2021). Genetic diversity and phylogeny of fleece-bearing goats of Central and Middle Asia. Russ. J. Genet..

[12] Voronkova V., Piskunov A., Nikolaeva E., Semina M., Konorov E., Stolpovsky Y.A. (2021). Haplotype Diversity of Mongolian and Tuvan Goat Breeds (*Capra hircus*) Based on mtDNA and Y-Chromosome Polymorphism. Russ. J. Genet..

[13] Porter V. (2020). Mason’s World Dictionary of Livestock Breeds, Types and Varieties.

[14] Takahashi H., Nyamsamba D., Mandakh B., Zagdsuren Y., Amano T., Nomura K., Yokohama M., Ito S.-I., Minezawa M. (2008). Genetic structure of Mongolian goat populations using microsatellite loci analysis. Asian-Australas. J. Anim. Sci..

[15] Chang C., Chow C., Tellier L., Vattikuti S., Purcell S., Lee J. (2015). Second-generation PLINK: Rising to the challenge of larger and richer datasets. Gigascience.

[16] Deniskova T.E., Dotsev A.V., Selionova M.I., Reyer H., Sölkner J., Fornara M.S., Aybazov A.-M.M., Wimmers K., Brem G., Zinovieva N.A. (2021). SNP-based genotyping provides insight into the West Asian origin of Russian local goats. Front. Genet..

[17] Berihulay H., Li Y., Liu X., Gebreselassie G., Islam R., Liu W., Jiang L., Ma Y. (2019). Genetic diversity and population structure in multiple Chinese goat populations using a SNP panel. Anim. Genet..

[18] DiveRsity: An R Package for the Estimation and Exploration of Population Genetics Parameters and Their Associated Errors-Keenan-2013-Methods in Ecology and Evolution-Wiley Online Library. https://besjournals.onlinelibrary.wiley.com/doi/10.1111/2041-210X.12067.

[19] Barbato M., Orozco-terWengel P., Tapio M., Bruford M.W. (2015). SNeP: A tool to estimate trends in recent effective population size trajectories using genome-wide SNP data. Front. Genet..

[20] Corbin L.J., Liu A.Y.H., Bishop S.C., Woolliams J.A. (2012). Estimation of historical effective population size using linkage disequilibria with marker data. J. Anim. Breed. Genet..

[21] Sved J.A., Feldman M.W. (1973). Correlation and probability methods for one and two loci. Theor. Popul. Biol..

[22] Meyermans R., Gorssen W., Buys N., Janssens S. (2020). How to study runs of homozygosity using PLINK? A guide for analyzing medium density SNP data in livestock and pet species. BMC Genom..

[23] Dunn.Test Function-Rdocumentation. https://www.rdocumentation.org/packages/dunn.test/versions/1.3.5/topics/dunn.test.

[24] Delaneau O., Marchini J., Zagury J.F. (2012). A linear complexity phasing method for thousands of genomes. Nat. Methods.

[25] Browning B.L., Browning S.R. (2013). Improving the accuracy and efficiency of identity-by-descent detection in population data. Genetics.

[26] Kamvar Z.N., Tabima J.F., Grünwald N.J. (2014). Poppr: An R package for genetic analysis of populations with clonal, partially clonal, and/or sexual reproduction. PeerJ.

[27] Raj A., Stephens M., Pritchard J.K. (2014). fastSTRUCTURE: Variational inference of population structure in large SNP data sets. Genetics.

[28] Behr A.A., Liu K.Z., Liu-Fang G., Nakka P., Ramachandran S. (2016). Pong: Fast analysis and visualization of latent clusters in population genetic data. Bioinformatics.

[29] Weir B.S., Cockerham C.C. (1984). Estimating F-statistics for the analysis of population structure. Evolution.

[30] Huson D.H., Bryant D. (2006). Application of Phylogenetic Networks in Evolutionary Studies. Mol. Biol. Evol..

[31] Bolormaa S., Ruvinsky A., Walkden-Brown S., Van der Werf J. (2008). Genetic relationships among Australian and Mongolian fleece-bearing goats. Asian-Australas. J. Anim. Sci..

[32] Meuwissen T.H. (2009). Accuracy of breeding values of’unrelated’individuals predicted by dense SNP genotyping. Genet. Sel. Evol..

[33] Ryman N., Laikre L., Hössjer O. (2019). Do estimates of contemporary effective population size tell us what we want to know?. Mol. Ecol..

[34] Green R.E., Gilbert G., Wilson J.D., Jennings K. (2020). Implications of the prevalence and magnitude of sustained declines for determining a minimum threshold for favourable population size. PLoS ONE.

[35] Islam R., Li Y., Liu X., Berihulay H., Abied A., Gebreselassie G., Ma Q., Ma Y. (2019). Genome-wide runs of homozygosity, effective population size, and detection of positive selection signatures in six Chinese goat breeds. Genes.

[36] Visser C., Lashmar S.F., Van Marle-Köster E., Poli M.A., Allain D. (2016). Genetic Diversity and Population Structure in South African, French and Argentinian Angora Goats from Genome-Wide SNP Data. PLoS ONE.

[37] Zheng Z., Wang X., Li M., Li Y., Yang Z., Wang X., Pan X., Gong M., Zhang Y., Guo Y. (2020). The Origin of Domestication Genes in Goats. Sci. Adv..

[38] Dmitriev N.G., Ernst N.K. Animal Genetic Resources of the USSR. https://www.fao.org/3/ah759e/ah759e00.htm.

