# Supplementary material for: Genetic Diversity, Population Structure and Phylogeny of Indigenous Goats of Mongolia Revealed by SNP Genotyping
Source: Animals (Basel). 2022 Jan 18;12(3):221. doi: 10.3390/ani12030221 (PMC8833718; doi:10.3390/ani12030221)
Supplement: Supplementary file 1 [file animals-12-00221-s001.zip › Supplementary figures S1-S4.pdf]

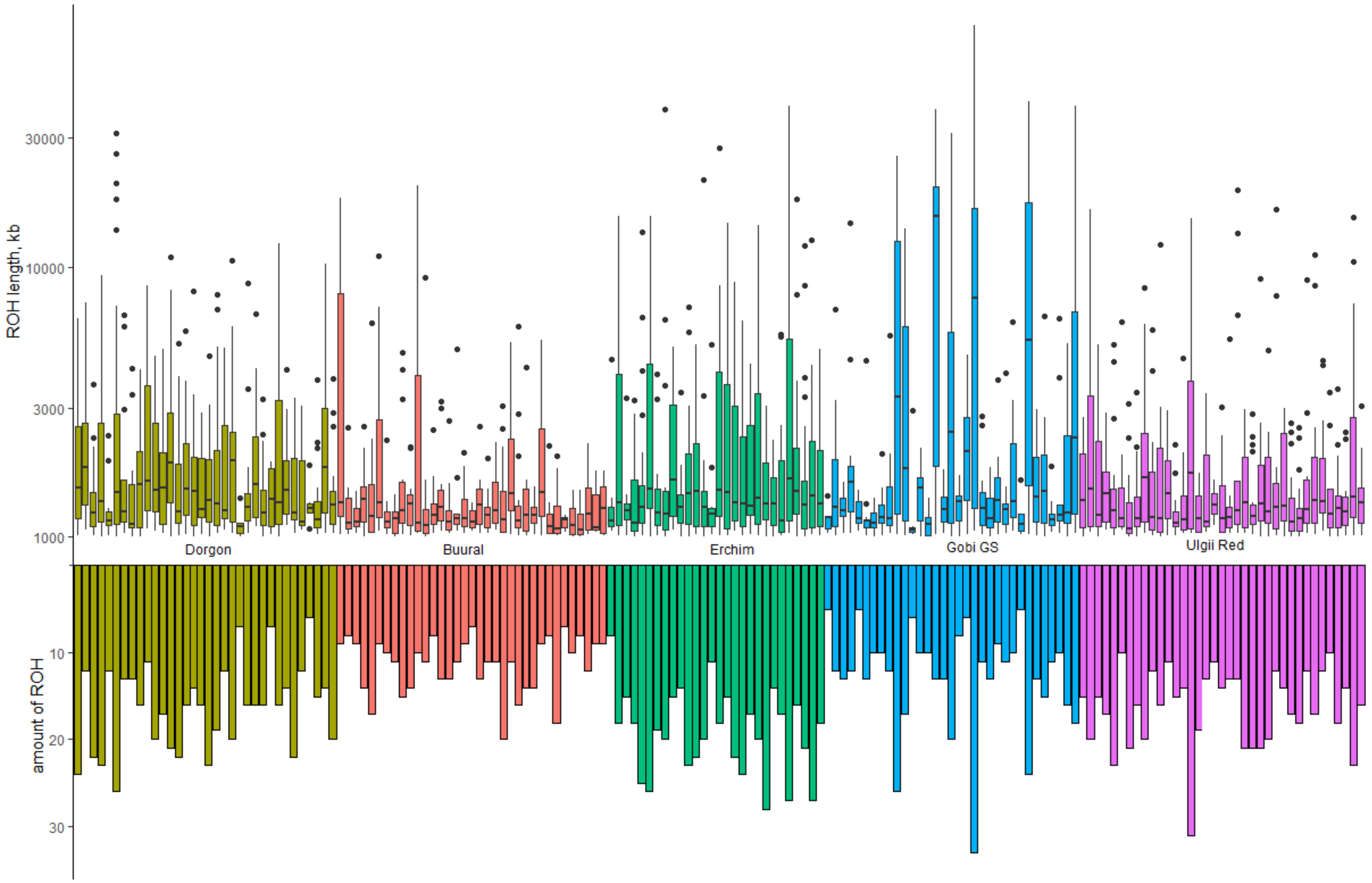

Figure S1. Distribution of ROH lengths (top) and amount of ROHs (down). Each bar represents one goat, colors mark breeds.

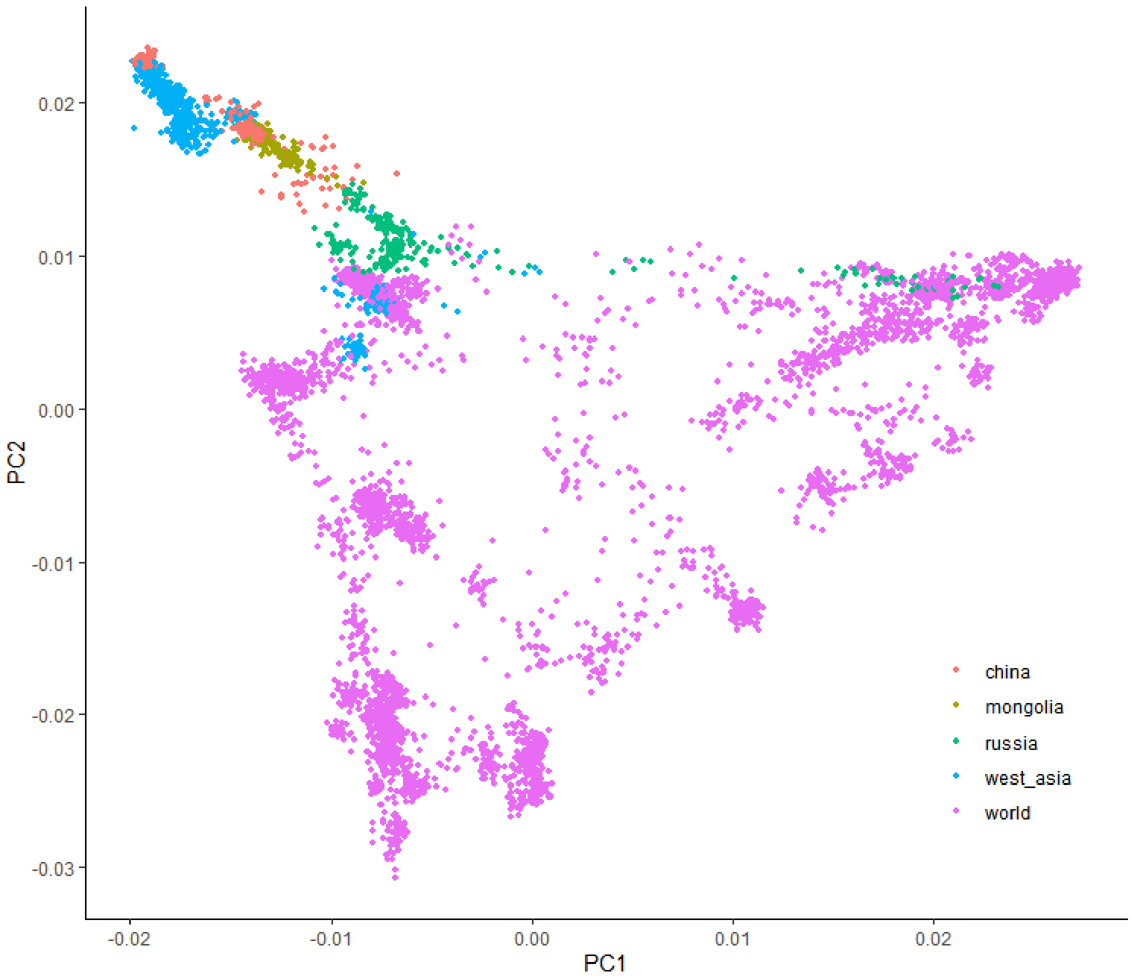

Figure S2. Principal component analysis for goat breeds from Mongolia, Russia, China, West Asia (Iran, Turkey and Pakistan), Europe, Africa, America and Australia

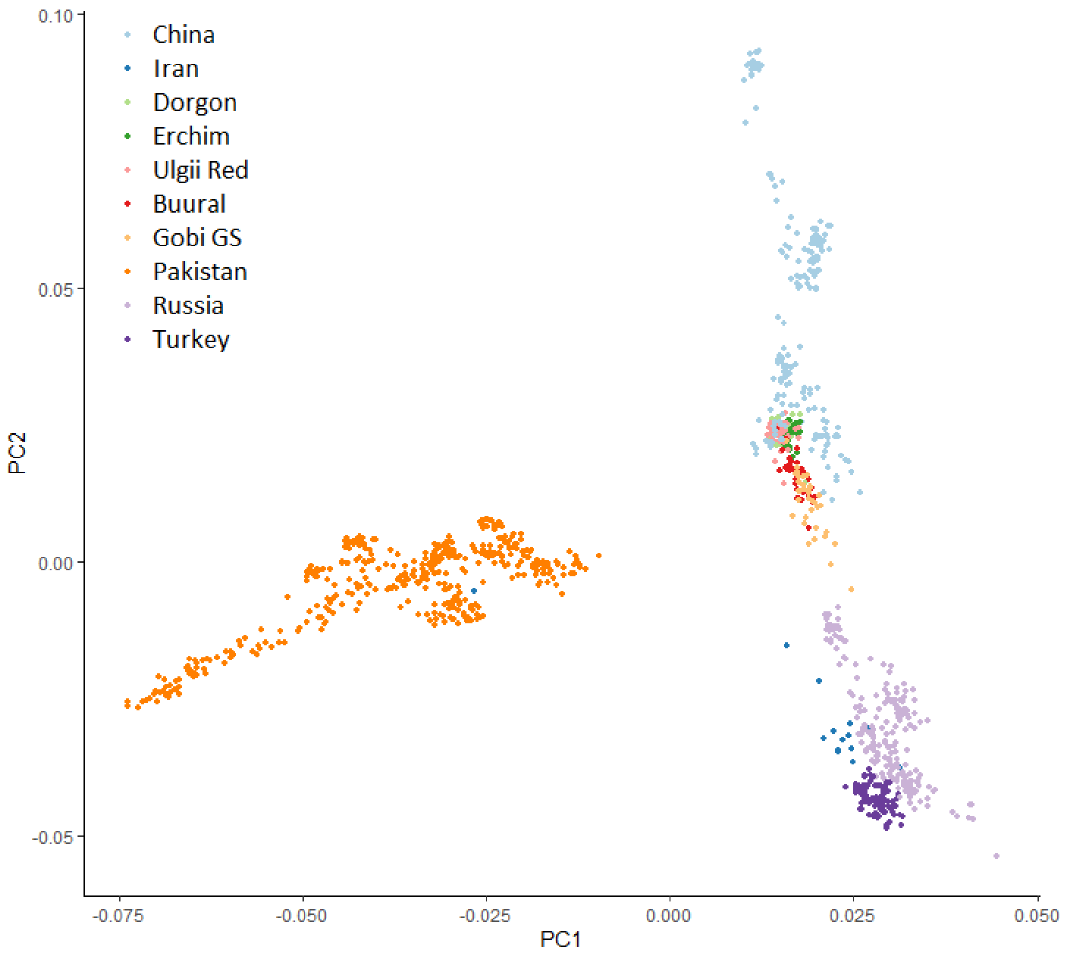

Figure S3. Principal component analysis for goat breeds from Mongolia, Russia, China,Iran, Turkey and Pakistan. Mongolian breeds are colored each separately and other breeds are colored by the country of their sampling.

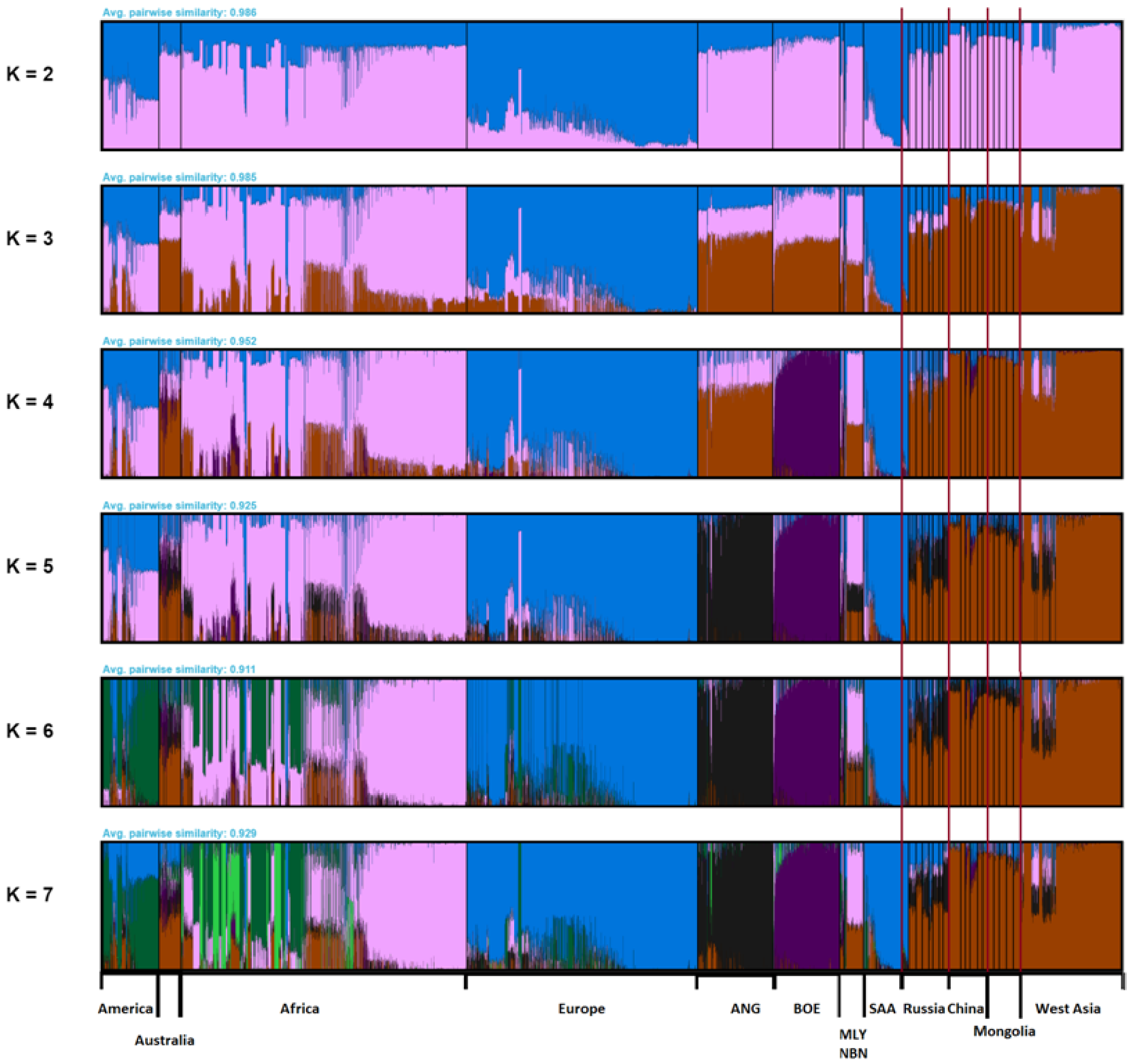

Figure S4. fastSTRUCTURE clustering for the worldwide dataset. Breeds are grouped by sampling location except large transborder Angora (ANG), Boer (BOE), Saanen (SAA) and two other breeds (Malayan (MLY) and Nubian (NBN)) sampled each in Tanzania/USA and Argentina/Egypt respectively.
